# Domain organization within the nuclear export factor Mex67:Mtr2 generates an extended mRNA binding surface

**DOI:** 10.1093/nar/gkv030

**Published:** 2015-01-23

**Authors:** Shintaro Aibara, Eugene Valkov, Meindert Lamers, Murray Stewart

**Affiliations:** MRC Laboratory of Molecular Biology, Francis Crick Avenue, Cambridge Biomedical Campus, Cambridge CB2 0QH, UK

## Abstract

The Mex67:Mtr2 complex is the principal yeast nuclear export factor for bulk mRNA and also contributes to ribosomal subunit export. Mex67 is a modular protein constructed from four domains (RRM, LRR, NTF2-like and UBA) that have been thought to be joined by flexible linkers like beads on a string, with the RRM and LRR domains binding RNAs and the NTF2-like and UBA domains binding FG-nucleoporins to facilitate movement through nuclear pores. Here, we show that the NTF2-like domain from *Saccharomyces cerevisiae* Mex67:Mtr2 also contributes to RNA binding. Moreover, the 3.3 Å resolution crystal structure of the Mex67^ΔUBA^:Mtr2 complex, supplemented with small angle X-ray scattering data, indicated that the LRR domain has a defined spatial relationship to the Mex67^NTF2L^:Mtr2 region. Conversely, the RRM domain and especially the UBA domain are more mobile. The conformation assumed by the LRR and NTF2-like domains results in clusters of positively-charged residues on each becoming arranged to form a continuous interface for binding RNA on the opposite side of the complex to the region that interacts with FG-nucleoporins to facilitate passage through nuclear pores.

## INTRODUCTION

The Mex67:Mtr2 complex (TAP:p15 or NXF1:NXT1 in metazoans) is the principal mRNA export factor in *Saccharomyces cerevisiae*. Mex67 is a member of the NXF family of proteins and has conserved homologs through eukaryotes from yeast to humans. Although sequence conservation is poor between *S. cerevisiae* Mex67 and *Homo sapiens* NXF1, they do show functional complementarity, in that over-expression of NXF1:NXT1 can rescue an otherwise lethal Mex67:Mtr2 deletion strain in *S. cerevisiae* (1). Mex67 and TAP/NXF1 are modular proteins that contain four structural domains: an N-terminal RNA recognition motif (RRM), a leucine rich repeat (LRR) domain, a nuclear transport factor 2-like domain (NTF2L) and an ubiquitin-associated domain (UBA). Mtr2/p15/NXT1 is a 15∼20 kDa protein that also has an NTF2-like fold and binds to the NTF2L domain of Mex67 (2,3).

In yeast, the Mex67:Mtr2 complex is thought to mediate the export of bulk mRNA through direct interactions with both the mRNA cargo and nuclear pore proteins that contain characteristic phenylalanine-glycine repeating sequence motifs (FG-nucleoporins or ‘FG-nups’) (3–5). A crystal structure of the RRM and LRR domains of *H. sapiens* NXF1 in complex with a fragment of the constitutive transport element (CTE-B), a viral RNA motif, confirmed that these two-N terminal domains are involved in binding cargo (6), whereas the NTF2L and UBA domains interact with a spectrum of FG-nucleoporins (3). Crystal structures have shown that the FG-containing cores of these motifs bind to surface hydrophobic patches on these two domains (7,8). The Mex67^NTF2L^:Mtr2 region has also been implicated in the export of pre-60S ribosomal subunits in *S. cerevisiae* (9,10).

Although high-resolution crystal structures of the individual NTF2L:Mtr2 region and UBA domains of Mex67:Mtr2 are available, the extent of sequence similarity to other NXF1 family members shown by the RRM domain is low and this has raised the possibility that Mex67 may differ from its metazoan counterparts in this region (11). Moreover, it is generally considered that the individual domains of Mex67 are joined by flexible linkers resulting in their being arranged like beads on a string and not having a defined spatial organization to one another. Here, we present a detailed structural analysis of the *S. cerevisiae* Mex67:Mtr2 complex using X-ray crystallography and small angle X-ray scattering (SAXS) that confirms the presence of the RRM and LRR domains as well as identifying a previously uncharacterized spatial relationship between the LRR and NTF2L domains that derives from interactions between Mtr2 and the linker between the LRR and NTF2L domains. We also show that RNA binding by the complex requires a contribution from the NTF2L domain in addition to the RRM and LRR domains. The alignment of the LRR and NTF2-like domains functions to generate an extensive positively-charged surface on the molecule that facilitates RNA binding and which positions the mRNA cargo on the opposite side of the nuclear export complex to that on which is located the sites that interact with FG-nucleoporins to mediate passage through nuclear pores.

## MATERIALS AND METHODS

### Cloning and protein purification

All constructs were cloned into the pETDuet-1 vector where Mex67 was cloned into the first multiple cloning site (MCS) and Mtr2 was cloned into the second MCS to generate various TEV cleavable His_6_ tagged constructs of Mex67:Mtr2. Domain boundaries were chosen based on crystal structures, resulting in the following constructs: Mex67 (residues 1–599, full length), Mex67^ΔUBA^ (residues 1–487), Mex67^LRR-NTF2L^ (residues 101–487), Mex67^NTF2L^ (residues 264–487) and Mex67^RRM-LRR^ (residues 2–262). All proteins were expressed in *Escherichia* *coli* BL21-CodonPlus(DE3)-RIL cells by IPTG induction at 18°C for 16 h. All purification steps and manipulations were conducted at 4°C unless stated otherwise.

Harvested cell pellets were resuspended in 5 ml of 50 mM Tris-HCl pH 8.0, 500 mM NaCl, 20 mM imidazole pH 8.0 per gram of wet cell pellet, after which the cells were lysed by high-pressure cavitation using 2 passes through an EmulsiFlex-C3 (AVESTIN). The lysate was clarified by centrifugation, and the supernatant incubated with Ni-NTA agarose beads for 1 h. Non-specifically bound proteins were removed by washing with 50 mM Tris-HCl pH 8.0, 500 mM NaCl, 20 mM imidazole pH 8.0. Bound protein was eluted with 50 mM Tris-HCl pH 8.0, 500 mM NaCl, 250 mM imidazole pH 8.0 and incubated with TEV protease overnight to remove the His_6_ tag. The sample was then diluted with 50 mM HEPES-NaOH pH 8.0 to reduce the NaCl concentration to ∼150 mM and purified with heparin affinity chromatography, using a 100 mM to 1 M NaCl gradient. The protein was then purified to homogeneity using a HiLoad 26/60 Superdex 200 column (GE Healthcare) in 20 mM HEPES-NaOH pH 8.0, 200 mM NaCl. Small aliquots of purified proteins were flash-frozen in liquid nitrogen in their final buffers and stored at -80°C until required.

### X-ray crystallography

Crystals of Mex67^ΔUBA^:Mtr2 were grown at 4°C by sitting-drop vapor diffusion in which 200 nl of purified protein was mixed with 200 nl of reservoir buffer (10% PEG 8000, 0.01 M zinc acetate, 0.1 M Tris-HCl pH 8.0). Crystals were obtained after 2–3 days and were harvested and cryo-cooled in mother liquor supplemented with 20% glycerol. X-ray diffraction data to a resolution of 3.3 Å were collected using beamline I04–1 at the Diamond Light Source (Oxford, UK), indexed and integrated using XDS (12), and reflections merged and scaled using AIMLESS (13,14). Molecular replacement using PHASER (15) and the *Chaetomium thermophilum* Mex67 LRR domain (PDB ID: 4WP6) and *S. cerevisiae* Mex67^NTF2L^:Mtr2 complex (PDB ID: 1OF5) as search models generated an initial model that, after iterative cycles of rebuilding using COOT (16) and refinement using PHENIX (17) generated a final model with an *R*_work_ of 21.6% (*R*_free_ = 28.3%), excellent geometry, and a MolProbity score (18,19) of 1.08 (100^th^ percentile). Detailed data collection and refinement statistics are given in Table 1.

**Table 1.** Crystal data

Symmetry	*P2_1_*
Unit cell dimensions	
*a, b, c* (Å)	101.0, 76.7, 199.0
α, β, γ (°)	90, 94.1, 90
***Data collection***	
Wavelength (Å)	0.9200
Resolution range (Å)^a^	49.7–3.30 (3.42–3.30)
Total observations^a^	163259 (17857)
Unique observations^a^	44836 (4787)
Completeness (%)^a^	97.4 (99.2)
Multiplicity^a^	3.6 (3.7)
*R*_pim_^a^	0.20 (0.86)
Mean I/σ(I)^a^	5.1 (1.3)
CC_1/2_^a^	95.7 (42.8)
Wilson *B* factor (Å^2^)	66.3
***Refinement***	
*R*_work_/*R*_free_ (%)	21.4/28.4
Number of atoms	
Protein (Non-H)	18797
Zinc ion	12
*B* factors (Å^2^)	
Protein atoms	54.5
Zinc ions	53.8
Geometry	
Bond length rmsd (Å)	0.004
Bond angle rmsd (°)	0.81
Ramachandran plot (%)	
favoured	98.1
allowed	1.9
forbidden	0
MolProbity score (19)/percentile	1.08/100

^a^Parentheses refer to final resolution shell.

### Small angle X-ray scattering

SAXS was performed using an on-line HPLC system (Viscotek) equipped with a Superdex 200 Increase 3.2/300 column (GE Healthcare) mounted on beamline BM29 at the European Synchrotron Radiation Facility (Grenoble, France). Data collection was conducted at 20°C, using a wavelength of 0.995 Å and a sample-to-detector distance of 1 m. Data were processed automatically by the on-line pipeline AUTOSUB (part of the ATSAS package (20)). Linear Guinier plots in the Guinier region (s*R_g_<1.3) were confirmed in all cases (Supplementary Figure S1). Pair distance distribution functions of the particles *P*(*r*) and the maximum sizes *D*_max_ were computed using GNOM (21) and molecular weights were estimated by comparison of the extrapolated forward scattering *I*(0) of the samples obtained using Guinier analysis by AUTORG (20) with that of a bovine serum albumin standard (Sigma-Aldrich). Calculation of theoretical SAXS profiles based on atomic models and comparisons with experimental SAXS profiles were conducted by submitting a PDB file and experimental data to the FoXS server (22,23). A summary of SAXS statistics is presented in Table 2.

**Table 2.** SAXS data

Protein construct	Theoretical Mw (kDa)	Estimated Mw (kDa)	Reciprocal space *Rg* (nm)	Real Space *Rg* (nm)	*D*_max_ (nm)
Mex67^LRR-NTF2L^:Mtr2	64.7	65.4	3.05	3.05	10.34
Mex67^ΔUBA^:Mtr2	75.9	78.3	3.58	3.58	12.79

### RNA binding assays

The interaction of RNA with a range of Mex67 constructs was assessed using fluorescence anisotropy assays in which 10 nM of DY-547 labeled homopolymeric RNA (A_15_, U_15_, C_15_, G_15_) was mixed with serially diluted protein in 20 mM Tris pH 8.0, 50 mM NaCl. Polarization readings were measured in a PHERAstar *Plus* microplate reader using the FP 540–20 590–20/590–20 module. Data analysis was conducted in the Prism software package and curves were fitted to the standard quadratic equation:

\begin{eqnarray*} &&{\rm Y} = {\rm mAU}_{\min } + \nonumber \\ &&\Delta {\rm mAU}_{\max } \left[ {\frac{{({\rm L} + {\rm K}_{\rm d} + {\rm X}) - \sqrt {({\rm L} + {\rm K}_{\rm d} + {\rm X})^2 - (4{\rm LX})} }}{{2{\rm L}}}} \right] \end{eqnarray*}

where Y is the observed anisotropy, mAU_min_ is the baseline anisotropy, ΔmAU_max_ is the maximum anisotropy change observed, L is the ligand concentration and X is the protein concentration.

## RESULTS

### Crystal structure of the Mex67^ΔUBA^:Mtr2 complex

**Figure 1.**
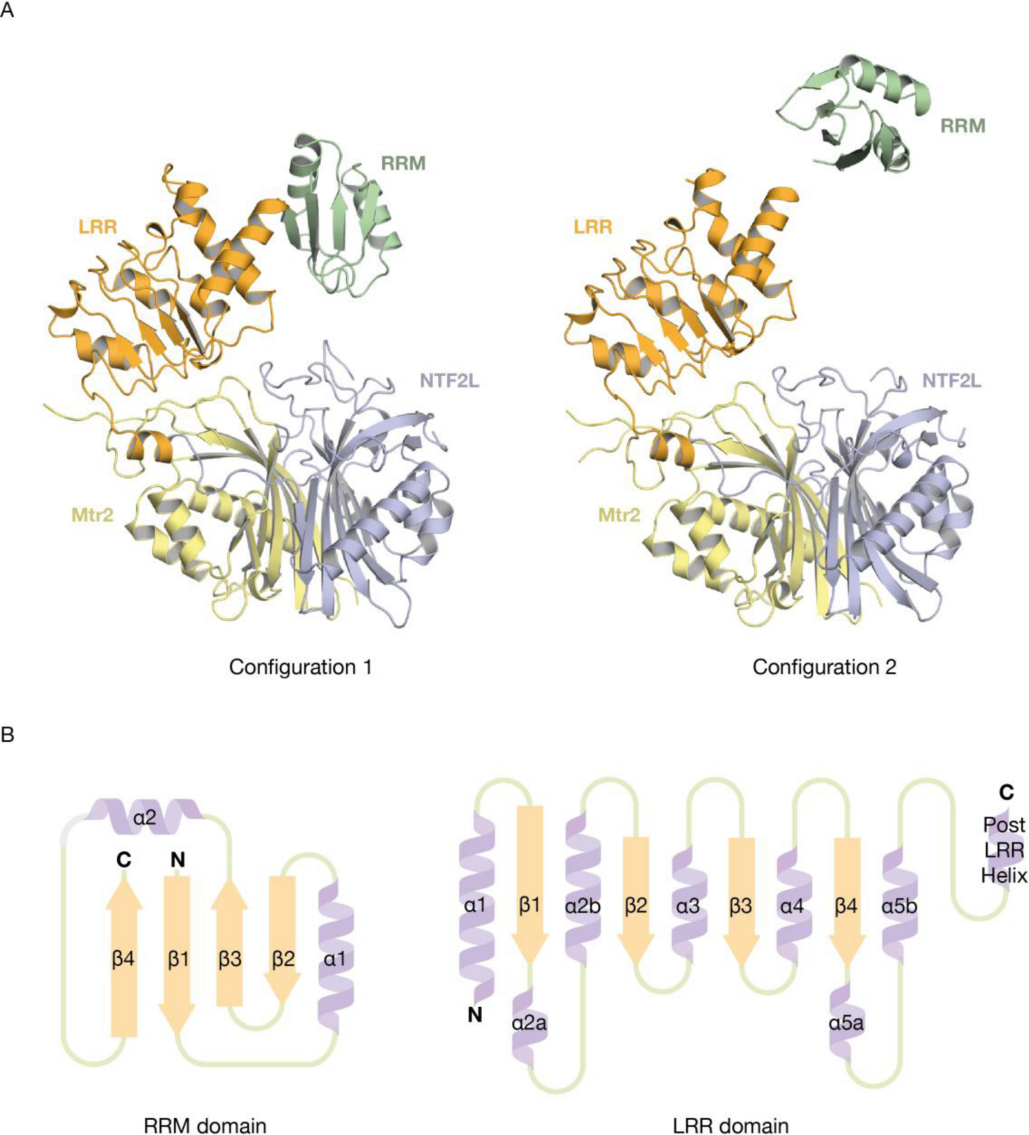
3.3 Å resolution crystal structure of Mex67^ΔUBA^:Mtr2. (**A**) All three domains (RRM, LRR and the NTF2L) of Mex67 and Mtr2 could be traced for the four copies in the asymmetric unit. Although all copies showed the same relative orientation of the LRR and NTF2L domains, the RRM domain was found in two different orientations (1 and 2), each of which was found in two copies related by pseudo-translational symmetry. (**B**) Schematic diagram of the secondary structure elements present in the RRM and LRR domains from *S. cerevisae* Mex67. The overall fold of the two domains is similar to that observed in *H. sapiens* NXF1.

Although extensive trials failed to yield crystals of full-length Mex67:Mtr2, diffraction quality crystals were obtained for a construct in which the UBA domain had been deleted (Mex67^ΔUBA^:Mtr2). These crystals had *P2_1_* symmetry and diffracted to 3.3 Å resolution using synchrotron radiation. Molecular replacement using the structure of the Mex67^NTF2L^:Mtr2 complex (PDB ID: 1OF5; (24)) together with the LRR domain of the *C. thermophilum* homologue (PDB ID: 4WP6) indicated that there were four copies of the protein complex in the asymmetric unit, arranged as two pairs related by pseudo-translational symmetry (Figure 1A). Although the RRM domain could not be located unequivocally using molecular replacement, electron density tentatively assigned to these missing domains was observed after several rounds of refinement and rebuilding (Supplementary Figure S2). A structural model of this region was obtained by manually docking the structure of the *C. thermophilum* Mex67 RRM domain (PDB ID: 4WPM) into this density, followed by rebuilding and refinement. After further cycles of refinement and rebuilding, a final model with an *R*_work_ of 21.4% (*R*_free_ = 28.4%) and excellent geometry was obtained (Table 1).

Although there was clear continuous electron density for the LRR and NTF2L domains and Mtr2 in the final *2Fo-Fc* maps, continuous density corresponding to the linker between the RRM and LRR domains was not observed, which prevented a completely unambiguous identification of each chain. Although it had previously been unclear whether *S. cerevisiae* Mex67 contained an RRM domain because the N-terminal region is ∼100 residues shorter when compared to metazoan homologues, the crystal structure clearly shows that a RRM domain is present, even though the sequence similarity is low (14% sequence identity over 70 residues when structurally aligned to *H. sapiens* NXF1^RRM^ using the DaliLite server (25)). Although the RRM domain displayed the characteristic βαββαβ-fold, it did not include the two typical RRM consensus sequence motifs (reviewed in (26)). The RRM consensus is comprised of two motifs: RNP1 (K/R)_o_-G-(F/Y)_o_-(G/A)_i_-F_o_-V_i_-X_o_-(F/Y)_i_ and RNP2 (L/I)_i_-(Y/F)_o_-(V/I)_i_-(G/N)_o_-(G/N)_o_-(L/M)_i_ where the subscripted ‘o’ or ‘i’ signify whether the side chain is surface exposed or facing the core of the RRM fold. In the case of *S. cerevisiae* Mex67, the structurally equivalent residues deviated from the consensus so that the RNP1 region had the sequence G-P_o_-L_o_-V_i_-I_o_-G-Y_o_-V_i_ and RNP2 had I_i_-S_o_-V_i_-R_o_-N_o_-W_i_. The LRR domain showed the characteristic pattern of alternating α-helices and β-strands arranged to form a gently curving structure similar to that observed in the *H. sapiens* NXF1 (11). There were no major structural differences between the *H. sapiens* NXF1 LRR and *S. cerevisiae* Mex67 LRR domains (Figure 1B and Supplementary Figure S3).

Strikingly, all four copies of the Mex67^ΔUBA^:Mtr2 complex in the asymmetric unit showed a conserved spatial arrangement of the LRR domain relative to the NTF2L domain. A dense network of interactions was observed between the region linking the LRR and NTF2L domains (the LRR-NTF2L linker) and specific regions of Mtr2. These additional interactions between Mex67 and Mtr2 (that are not part of the canonical NTF2-like interactions between the β-sheets of the NTF2L domain and Mtr2) appeared to contribute to the spatial arrangement of the LRR and NTF2L domains. In contrast to the defined spatial orientation of the LRR domain and the NTF2L:Mtr2 region, the position taken up by the RRM domain relative to the LRR domain appeared to be dictated primarily by lattice contacts and so, although a spatial arrangement between the RRM domain and the LRR domain was observed in the NXF1^RRM-LRR^:CTE-B RNA structure (6), this appears to have been primarily a result of the RRM and LRR domains being bound to the structured CTE-B RNA.

### Contribution of the NTF2L domain pre-α1 loop and Mtr2 to LRR positioning

**Figure 2.**
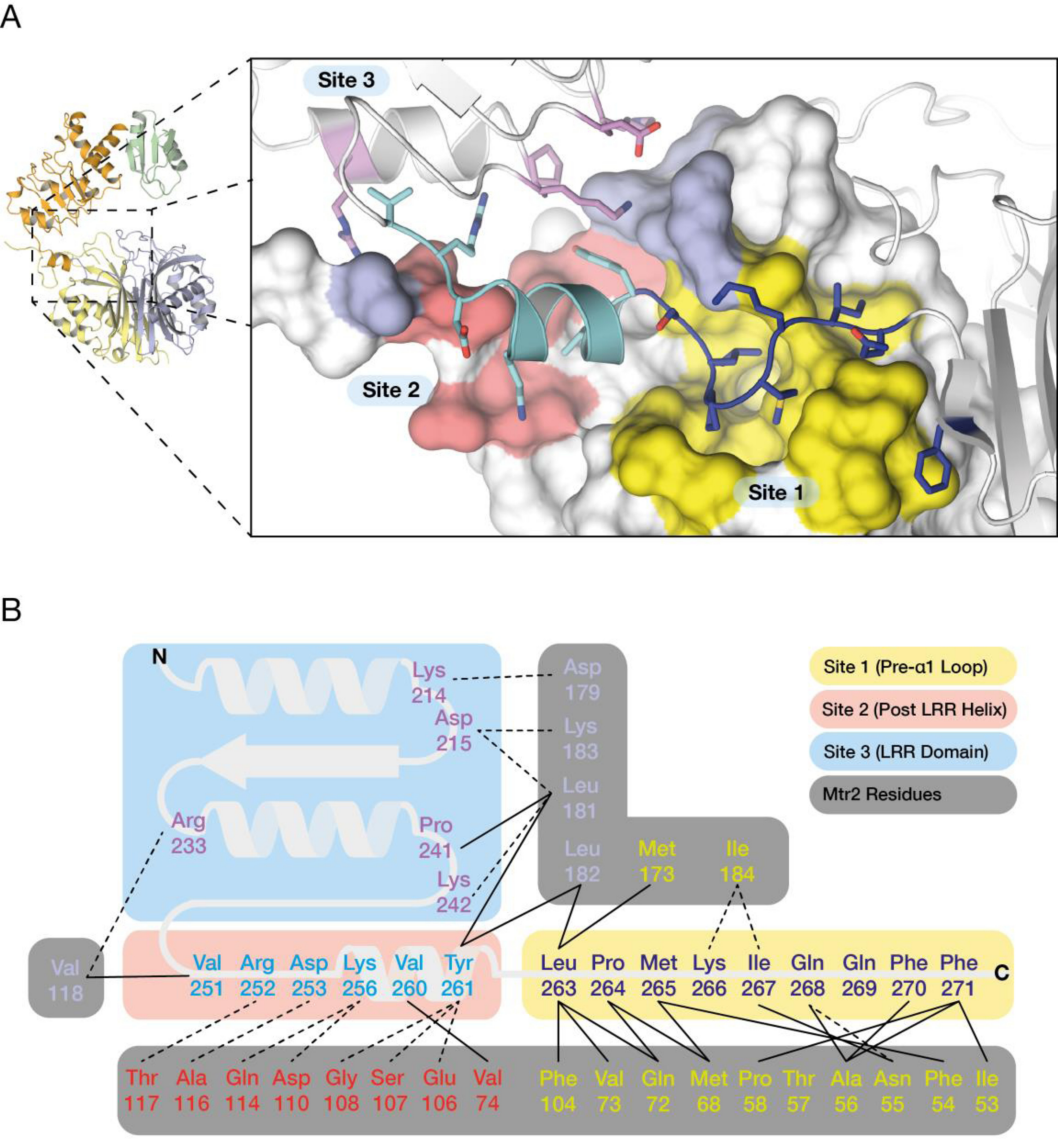
Interface formed between the LRR domain and NTF2L:Mtr2. (**A**) The extensive interaction surface can be divided into three sites: Site 1 (yellow), where the pre-α1 loop of the NTF2L domain interacts with the Mtr2 core; Site 2 (pink), where a short helical segment after the LRR domain interacts with the internal loop of Mtr2; and site 3 (blue) where LRR residues interact with Mtr2. (**B**) Schematic representation of the network of interactions between Mex67 and Mtr2 excluding the NTF2L core. Broken lines represent putative H-bonds and solid lines represent hydrophobic interactions.

The principal interaction surface between Mex67-NTF2L and Mtr2 was extensive, with 1963 Å^2^ being buried by the canonical NTF2-like interactions between the curved β-sheets of the NTF2L domain and Mtr2. However, in the present crystal structure, an additional interface was observed that was formed by regions outside the NTF2-like core and which buried 1208 Å^2^ between Mex67 and Mtr2 (Figure 2A). This interface was dominated by interactions between the LRR-NTF2L linker of Mex67 that was buried in a shallow groove that ran across the surface of Mtr2 and was supplemented by a series of H-bonds and salt bridges between the LRR domain and Mtr2 as illustrated schematically in Figure 2B. The interaction between the LRR-NTF2L linker involved mainly hydrophobic interactions between the loop region immediately prior to the first helix (α1) of the NTF2L domain (pre-α1 loop) and a hydrophobic channel on the surface of Mtr2 (site 1, blue on yellow in Figure 2) where Leu263, Met265 and Phe271 of Mex67 made major contributions to this interaction. This interaction was supplemented by interactions involving primarily putative salt bridges and/or H-bonds deriving from residues located in a short helical segment at the C-terminus of the LRR domain with the *S. cerevisiae-*specific internal loop on the surface of Mtr2 (site 2, cyan on red in Figure 2) and a group of residues in consecutive loops near the middle of the LRR domain (site 3, purple on light blue in Figure 2).

**Figure 3.**
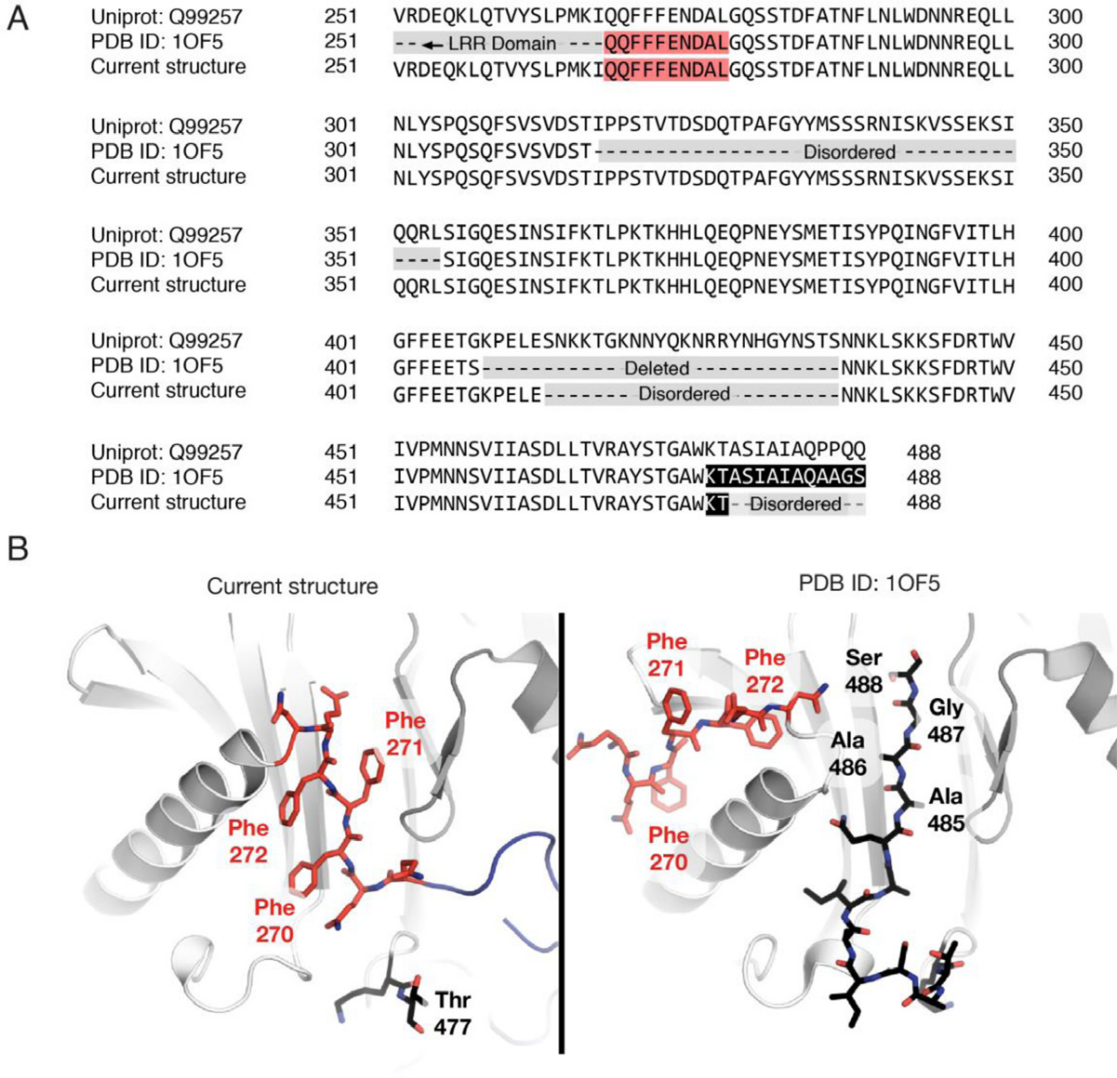
Differences in the position of the pre-α1 loop between the Mex67^NTF2L^:Mtr2 complex in the multi-domain structure and the structure of the individual domain obtained previously ((8); PDB ID: 1OF5). (**A**) Sequence differences between the constructs employed to obtain the different structures of Mex67^NTF2L^:Mtr2. In the multi-domain structure it was possible to trace residues 317–354 that were previously disordered, although an internal loop that was previously deleted in the construct used to obtain the structure of the isolated domain (24) was also disordered in this structure. The extreme C-terminal residues present in the isolated domain structure differ from the native sequence of the protein. (**B**) View of the pre-α1 loop. In the previous structure (24), the non-native residues (AAGS, residues 485–488) introduced at the C-terminus of the NTF2L domain formed αβ−strand that sterically inhibited the pre-α1 loop from adopting the native conformation. The triple Phe motif present in the pre-α1 loop (residues 270–272) formed distinct contacts with both the NTF2-like core and Mtr2, resulting in the loop adopting a considerably different conformation (shown in red and labeled).

Although the structure of the NTF2L:Mtr2 region in the present structure closely resembled that in Mex67^NTF2L^:Mtr2 complex alone (24), it was possible to trace several loop regions that were disordered in the earlier structure. Thus, there was electron density corresponding to the ‘internal’ loop present in Mtr2 between strands β4 and β5 that could be traced over most of its length (Figure 3 and Supplementary Figure S4) and which is involved in interactions with the LRR domain described in detail above (sites 2 and 3, see Figure 2). The position of residues 268–276 that form part of the pre-α1 loop was strikingly different between the present structure and that determined previously (PDB ID: 1OF5; (24)). This difference was probably due to the previous crystal structure being obtained from a construct in which Mex67 residues 485–488 had been mutated from PPQQ to AAGS (Figure 3A and Supplementary Figure S4) that resulted in their forming a short β-strand in the position occupied by the pre-α1 loop in the present structure. The proline-rich native sequence of this C-terminal region of the NTF2L domain is unlikely to form a regular secondary structure and was disordered in the present structure, which was critical in allowing the pre-α1 loop to be positioned close to Mtr2 and interact intimately with it (Figure 3B).

**Figure 4.**
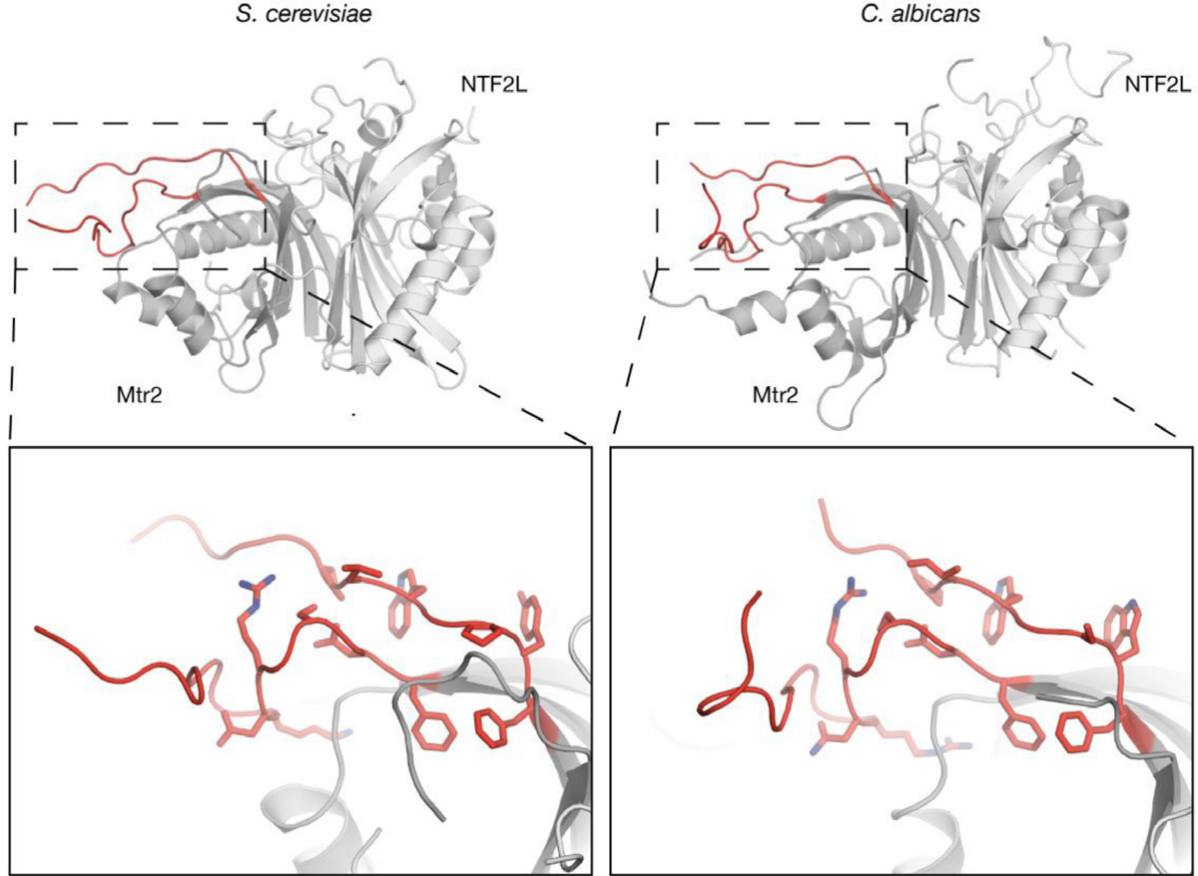
Structural comparison of the Mtr2 internal loop between *S. cerevisiae* and *C. albicans*. Mtr2 proteins from lower eukaryotes such as *S. cerevisiae* and *C. albicans* have large internal loops, whereas higher metazoans do not. Comparison of the two internal loops present on *S. cerevisiae* and *C. albicans* Mtr2 shows conserved features that give rise to the distinct loop associated with the export of ribosomal subunits (10). It is likely that *C. albicans* Mex67:Mtr2 can form the same domain arrangement as *S. cerevisiae* Mex67:Mtr2.

The pre-α1 loop was also present in the structure of the *H. sapiens* NXF1^NTF2L^:NXT1 complex ((8); PDB ID: 1JKG), where it was placed in a position very similar to that seen in the present structure. Although *H. sapiens* NXF1-Leu370, that is in the structurally equivalent position to *S. cerevisiae* Mex67-Leu263, was mutated to alanine in the *H. sapiens* NXF1^NTF2L^:NXT1 structure, the overall placement of the pre-α1 loop is consistent with this interaction occurring. Although unfortunately the pre-α1 loop was omitted from the constructs used to obtain structures of the equivalent regions from the *Candida albicans* ((27); PDB ID: 1Q40,) and *Caenorhabditis elegans* ((28); PDB ID: 3NV0) proteins, these structures retained the hydrophobic channel on the surface of Mtr2 and furthermore, *C. albicans* Mtr2 retained a large partially ordered internal loop that is similar to that observed in the present *S. cerevisiae* structure, and so could potentially interact with the LRR domain in a similar fashion to that observed here (Figure 4).

**Figure 5.**
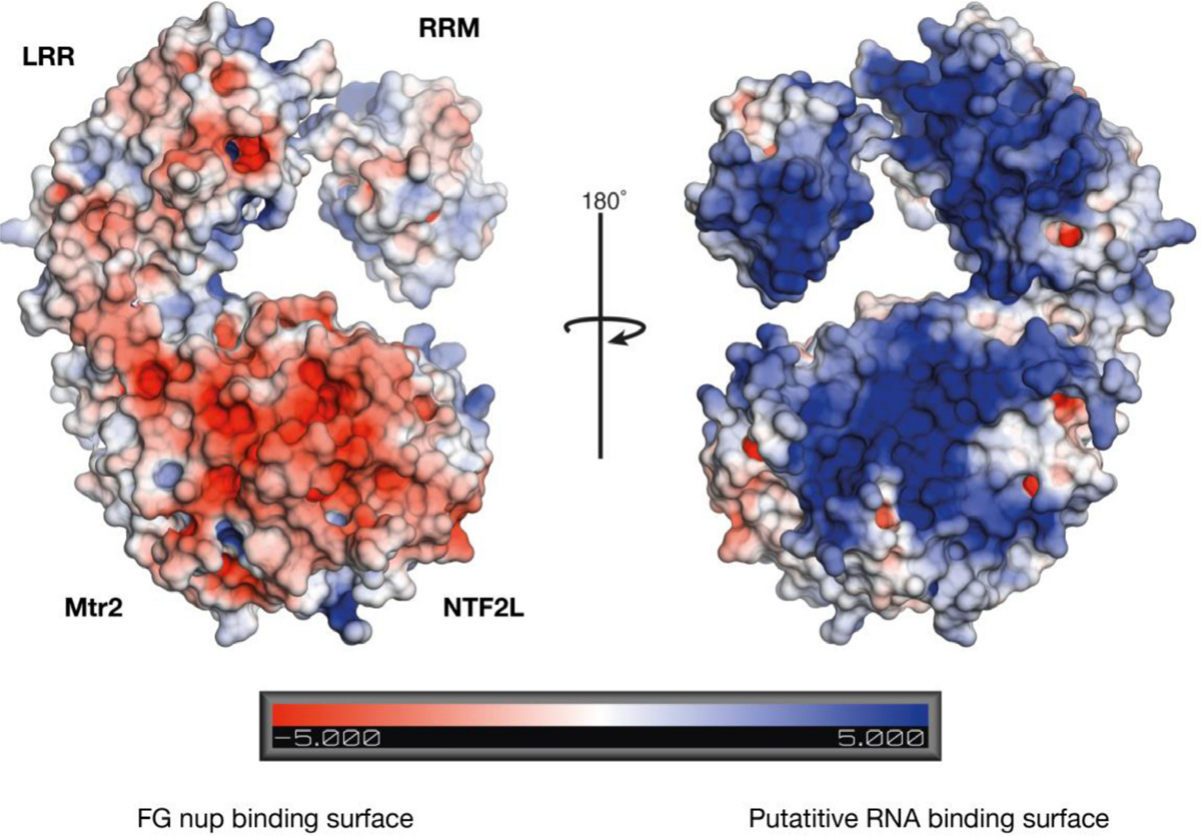
Electrostatic surface potential of the Mex67^ΔUBA^:Mtr2 complex. The surface potential showed a biased distribution in which the side on which the FG-nucleoporin binding site is located was more negatively charged, whereas the opposite side of the complex had a large continuous band of positive charge that derived from all the domains. Importantly, the positively charged surface formed by the RRM and LRR domain also extended into the NTF2L domain and Mtr2, consistent with all these domains contributing to a continuous RNA binding interface. The electrostatic surface potential displays a range of ± 5 kT/e, where deep blue represents positive and red represents negatively charged areas. The electrostatic potentials were calculated by solving the Poisson–Boltzmann equation with the APBS plugin in PyMOL using the default settings.

Overall, the arrangement of the three Mex67 domains formed a rather flat and compact molecule, in which the domains were placed in the same plane. The electrostatic potential calculations of Mex67^ΔUBA^:Mtr2 indicated that one side of the molecule was more positively charged than the other (Figure 5). Furthermore, the nucleoporin binding site previously identified in the human homologue was located on the opposite, negatively charged surface. This arrangement raised the possibility that the positively charged surface could function as a large RNA binding surface in which the NTF2L domain and Mtr2 also contributed to the interaction, while leaving the FG-nucleoporin binding site exposed on the opposite side of the molecule and free to interact with FG-nucleoporins.

### SAXS supports the LRR-NTF2L arrangement

**Figure 6.**
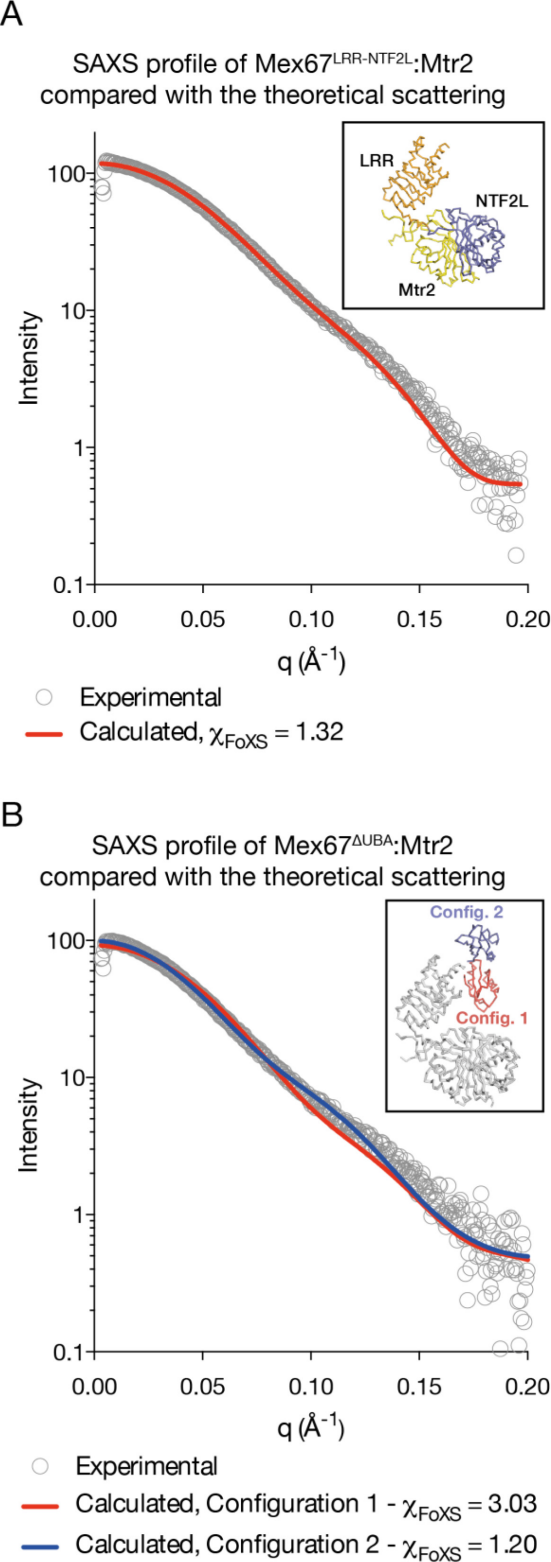
Small-angle X-ray scattering data. (**A**) The theoretical scattering of a model for the Mex67^LRR-NTF2L^:Mtr2 based on the crystal structure is in good agreement with the measured SAXS profile. (**B**) Configuration 2 observed in the crystal structure of Mex67^ΔUBA^:Mtr2 is in excellent agreement with the SAXS profile. Both SAXS profiles support the hypothesis that there is a stable arrangement between the LRR and NTF2L domain of Mex67. The atomic model submitted to the FoXS server to calculate the theoretical scattering is shown within a box in each panel.

SAXS was employed to probe whether the domain arrangement observed in the crystal structure of Mex67^ΔUBA^:Mtr2 was also present in solution. Because multiple arrangements of the RRM domain were present in the crystal structure, a construct that contained only the LRR and NTF2L domains together with Mtr2 was used initially. A model for Mex67^LRR-NTF2L^:Mtr2 was generated by omitting the RRM domain from the crystal structure of Mex67^ΔUBA^:Mtr2 and had a theoretical scattering calculated using the FoXS server that matched the experimental data well (χ_FoXS_ = 1.32, Figure 6A).

The SAXS profile collected for Mex67^ΔUBA^:Mtr2 was also compared against a range of atomic models. Consistent with the RRM-LRR linker region being flexible, multiple arrangements of the RRM domain gave a superior fit to the measured SAXS profile (Figure 6B). Configuration 2 from the crystal structure of Mex67^ΔUBA^:Mtr2 produced an excellent fit with χ_FoXS_ = 1.20. On the other hand, Configuration 1 fit less well with χ_FoXS_ = 3.03. An atomic model completely omitting the RRM domain (equivalent to the model used for Mex67^LRR-NTF2L^: Mtr2) produced the worst fit with χ_FoXS_ = 3.74 (data not shown).

### The NTF2L domain and Mtr2 both contribute to binding RNA

**Figure 7.**
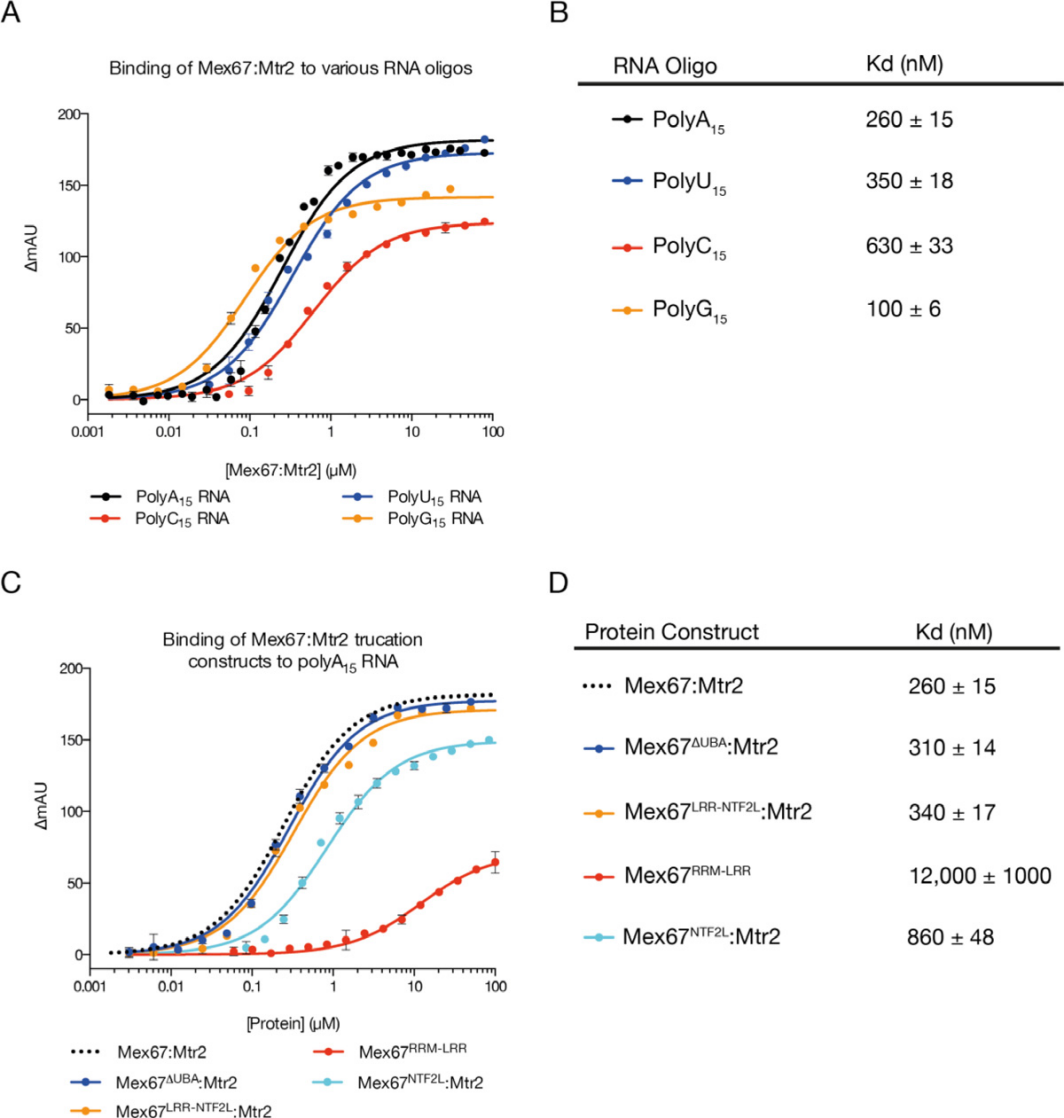
RNA binding affinities. (**A** and **B**) Fluoresence anisotropy assays conducted using Mex67:Mtr2. Data were fitted to the standard quadratic binding equation to obtain Kd values. All four tested RNA oligonucleotides tested bound, albeit with different affinities. Consistent with previous findings, Mex67:Mtr2 binds polyA_15_ and polyG_15_ more tightly than polyU_15_ and polyC_15_ (2). (**C** and **D**) Deletion of NTF2L:Mtr2 domains from the Mex67:Mtr2 complex reduces the affinity for polyA_15_ RNA by ∼40-fold. On the other hand, deletion of the RRM and LRR domain reduces the binding affinity by only ∼3-fold, highlighting the importance of the NTF2L:Mtr2 region for RNA binding. Curves reproduced from panel A are shown as dotted lines without the data points.

The way in which the spatial arrangement of the LRR and NTF2L domains and Mtr2 generated a large positively-charged surface on Mex67 indicated that the NTF2L domain might contribute to RNA binding, even though previously this function had thought to reside in the RRM and LRR domains. Consistent with previous RNA binding studies (2), although Mex67:Mtr2 bound A_15_, U_15_, C_15_ and G_15_ RNA polynucleotides, A_15_ and G_15_ RNA were bound more tightly than U_15_ and C_15_ RNA (Figure 7A and B). Because G-rich RNA sequences frequently adopt higher order structures, potentially producing aptamers, A_15_ RNA was employed as the substrate for subsequent assays. To achieve binding affinity for A_15_ RNA comparable to the full four-domain Mex67:Mtr2 construct, the first three domains (RRM-LRR-NTF2L) together with Mtr2 were required (Figure 7C and D). Thus a 260 nM interaction with A_15_ RNA was observed when all four domains of Mex67 plus Mtr2 were present and binding was not impaired by removal of the UBA domain, so that Mex67^ΔUBA^:Mtr2 retained comparable affinity (310 nM). Intriguingly, the Mex67^RRM-LRR^ construct, in which the NTF2L:Mtr2 region had been removed, had a much weaker affinity of 12 μM, even though it retained the domains previously thought to be primarily involved in binding RNA. Conversely, deletion of the RRM domain (resulting in the Mex67^LRR-NTF2L^:Mtr2 construct) produced only a marginal decrease in affinity for A_15_ RNA (340 nM) compared to the Mex67^ΔUBA^:Mtr2 construct (310 nM). The observation that the Mex67^RRM-LRR^ construct showed comparatively weak affinity for A_15_ RNA whereas the Mex67^LRR-NTF2L^:Mtr2 showed only a marginal decrease indicated that the NTF2L domain in complex with Mtr2 itself might bind RNA and indeed was shown to bind A_15_ RNA with 860 nM affinity.

**Figure 8.**
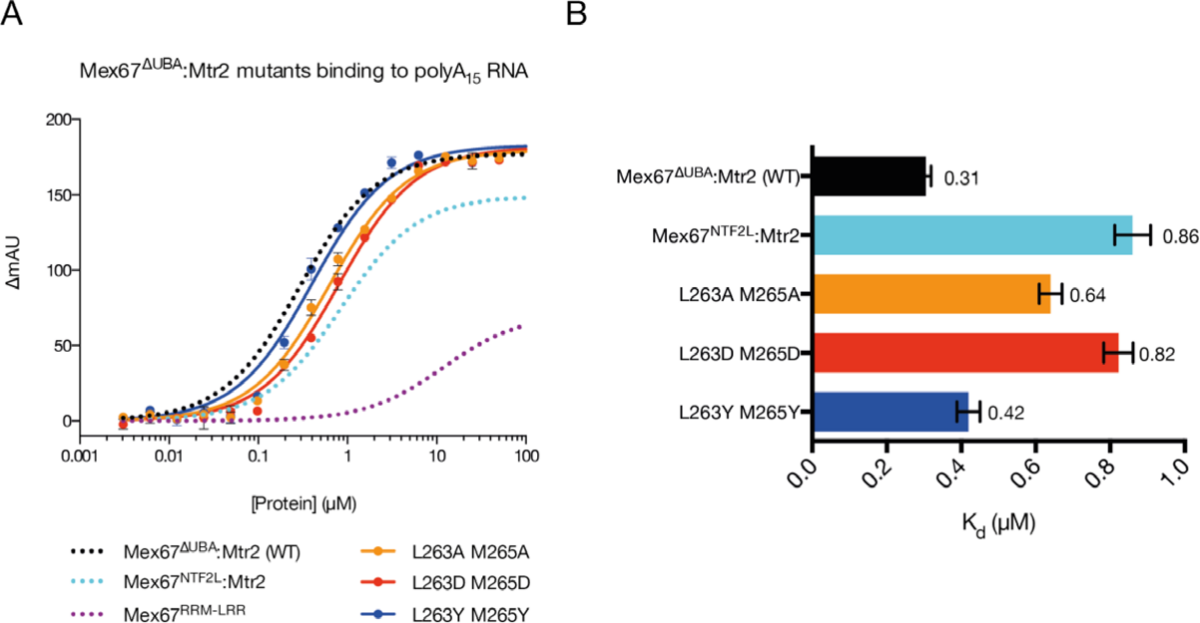
Mutations in the pre-α1 loop of the NTF2L domain of Mex67 reduce the binding affinity to polyA_15_ RNA. Fluoresence anisotropy assays (**A**) and a comparison of the Kd values obtained for Mex67^ΔUBA^:Mtr2 mutants binding to polyA_15_ RNA (**B**). The greatest decreases in affinity were observed by substitutions to Asp when the binding affinity exhibited by Mex67^ΔUBA^:Mtr2 was reduced to a level comparable to a deletion of the RRM and LRR domains. Curves reproduced from Figure 7C are shown as dotted fits without the data points.

The hypothesis that the spatial positioning of the LRR domain relative to NTF2L:Mtr2 contributed to a continuous RNA binding interface was tested by employing mutagenesis to impair the interaction between the domains (Figure 8). Two residues (Leu263 and Met265) that the crystal structure indicated were central to the interaction between the pre-α1 loop and Mtr2 were mutated to Ala, Asp or Tyr. These double mutants retained the ability to bind Mtr2, indicating that the NTF2-like fold remained intact. These mutations altered affinity of Mex67^ΔUBA^:Mtr2 for A_15_ RNA to varying extents. The greatest reduction in affinity was seen with mutation to Asp (L263D + M265D) that introduced charged residues into a principally hydrophobic interface, and which reduced the affinity of Mex67^ΔUBA^:Mtr2 for A_15_ RNA from 310 nM (wild-type) to 820 nM (L263D + M265D). This reduced affinity for A_15_ RNA was comparable to that seen with the NTF2L:Mtr2 region alone (that is with the RRM and LRR domains deleted) where an affinity of 860 nM was observed. Ala and Tyr substitutions exhibited a reduced impact on the affinity (640 nM and 420 nM respectively) probably because they were less efficient at disrupting the interaction interface that was dominated by hydrophobic contacts in this region.

## DISCUSSION

Both crystallography and SAXS have identified a previously uncharacterized spatial organization within the Mex67:Mtr2 complex in which the LRR domain has a defined orientation relative to the NTF2L:Mtr2 region that is generated primarily by an interaction between Mtr2 and the LRR-NTF2L linker. A conserved interaction between the pre-α1 loop of the NTF2L domain and Mtr2 appears to be important for this domain organization, with additional interactions formed between the LRR domain and Mtr2. This spatial arrangement generates a large, positively-charged, surface on one side of the molecule in which the NTF2L domain augments the contributions made to RNA binding by the RRM and LRR domains.

The demonstration that Mex67^NTF2L^:Mtr2 interact directly with RNA in the absence of the RRM and LRR domains, together with the results of the range of truncation mutants in which individual domains were removed (Figure 7C and D) indicated that the NTF2L:Mtr2 region forms an integral component of the interface on Mex67:Mtr2 that binds RNA. Moreover, both the crystal structure of Mex67^ΔUBA^:Mtr2 and SAXS data indicated that these three domains can be arranged to generate a continuous positively-charged surface on one face of the molecule that would be ideally suited to generating a RNA binding interface spanning all three domains (Figure 5). This hypothesis was supported by the decrease in affinity for A_15_ RNA seen with mutants designed to decouple the continuous RNA binding interface by impairing the interaction between the LRR-NTF2L linker and Mtr2 that is important for maintaining their spatial arrangement. Because these mutations were located in the linker region they were unlikely to interfere with the fold of either domain and this was supported by these mutant Mex67 chains retaining Mtr2 binding. The introduction of charged residues (L263D + M265D) into the mainly hydrophobic interface between the linker and Mtr2 reduced the affinity of the Mex67^ΔUBA^:Mtr2 complex for RNA to levels comparable to a construct only including Mex67^NTF2L^:Mtr2 alone, consistent with the spatial arrangement of the LRR and NTF2L domains contributing to the RNA binding interface. These mutations were in regions of the Mex67:Mtr2 complex previously thought to be unrelated to RNA binding. Both Leu263 and Met265 reside on the negatively charged side of the structure, where the FG-nucleoporin binding site is also located (8). Moreover, in the crystal structure of the Mex67^ΔUBA^:Mtr2 complex, both residues are buried in a hydrophobic channel on Mtr2 and so are unlikely to be involved directly with RNA binding.

In yeast, such as *S. cerevisiae* and *C. albicans*, Mtr2 has a long internal loop present between strands β4 and β5. Although this loop has been proposed to be important in pre-60S ribosomal subunit export by contributing to an extended binding interface (10), the results presented here raise possibility that disrupting this loop in Mtr2 could alter the overall domain organization within the Mex67:Mtr2 complex. Thus this Mtr2 internal loop could have a dual function by forming a binding surface for large RNA substrates by maintaining the spatial arrangement of the LRR and NTF2L domains as well as forming direct interactions with RNA, at least in ribosomal subunits. Although the NTF2L domain also has an equivalent internal loop between strands β4 and β5 that has also been proposed to be involved in ribosomal subunit export, this loop was disordered in our structure.

In contrast to the defined spatial relationship observed between the Mex67 LRR and NTF2 domains, in both the crystal structure and in solution (monitored by SAXS), the position of the RRM domain appeared to be more variable (Supplementary Figure S5) and did not correspond to that observed in the crystal structure of CTE-RNA complexed with the RRM-LRR region of *H. sapiens* NXF1 (6). It is likely that the linker between the RRM and LRR domains is sufficiently flexible to enable the position of RRM domain to move to accommodate binding partners such as mRNA or, in the crystal unit cell, form weak crystal packing contacts with adjacent chains. Because of the differences in the mRNA export pathway between yeast and metazoans (29), further work will be required to establish the extent to which the results obtained here for Mex67 can be extended to NXF1, although, since these molecules all have similar structures, it is likely that they recognize mRNAs in similar ways.

In summary, the crystal structure of the *S. cerevisiae* Mex67^ΔUBA^:Mtr2 complex has shown an unanticipated spatial arrangement of the LRR and NTF2L domains that generates a large positively-charged surface on one side of the complex that is ideally positioned to form a continuous RNA binding surface. Moreover, in addition to facilitating nuclear export through interactions with FG-nucleoporins, the NTF2L domain of Mex67 also makes an important contribution to the affinity of the complex for RNA in addition to the previously characterized binding activity of the RRM and LRR domains.

## ACCESSION NUMBERS

Coordinates and structure factors for the 3.3 Å resolution Mex67^ΔUBA^:Mtr2 complex have been deposited in the Protein Data Bank with accession number 4WWU.

## SUPPLEMENTARY DATA

Supplementary Data are available at NAR Online.

SUPPLEMENTARY DATA
