# Supplementary material for: Domain organization within the nuclear export factor Mex67:Mtr2 generates an extended mRNA binding surface
Source: Nucleic Acids Res. 2015 Jan 23;43(3):1927–36. doi: 10.1093/nar/gkv030 (PMC4330389; doi:10.1093/nar/gkv030)
Supplement: SUPPLEMENTARY DATA [file supp_43_3_1927__index.html]

Domain organization within the nuclear export factor Mex67:Mtr2 generates an extended mRNA binding surface — SUPPLEMENTARY DATA 

# Domain organization within the nuclear export factor Mex67:Mtr2 generates an extended mRNA binding surface

## SUPPLEMENTARY DATA

**Files in this Data Supplement:**

- SUPPLEMENTARY DATA
