# Supplementary material for: Domain organization within the nuclear export factor Mex67:Mtr2 generates an extended mRNA binding surface
Source: Nucleic Acids Res. 2015 Jan 23;43(3):1927–36. doi: 10.1093/nar/gkv030 (PMC4330389; doi:10.1093/nar/gkv030)
Supplement: SUPPLEMENTARY DATA [file supp_gkv030_nar-03305-r-2014-File010.pdf]

SUPPLEMENTAL INFORMATION

Domain organization within the nuclear export factor Mex67:Mtr2 generates an extended mRNA binding surface

Shintaro Aibara, Eugene Valkov, Meindert Lamers and Murray Stewart*

MRC Laboratory of Molecular Biology, Francis Crick Avenue, Cambridge Biomedical Campus, Cambridge CB2 0QH, UK.

*Author for correspondence. Phone +44 1223 267-74; e-mail: ms@mrc-lmb.cam.ac.uk

SUPPLEMENTARY FIGURES

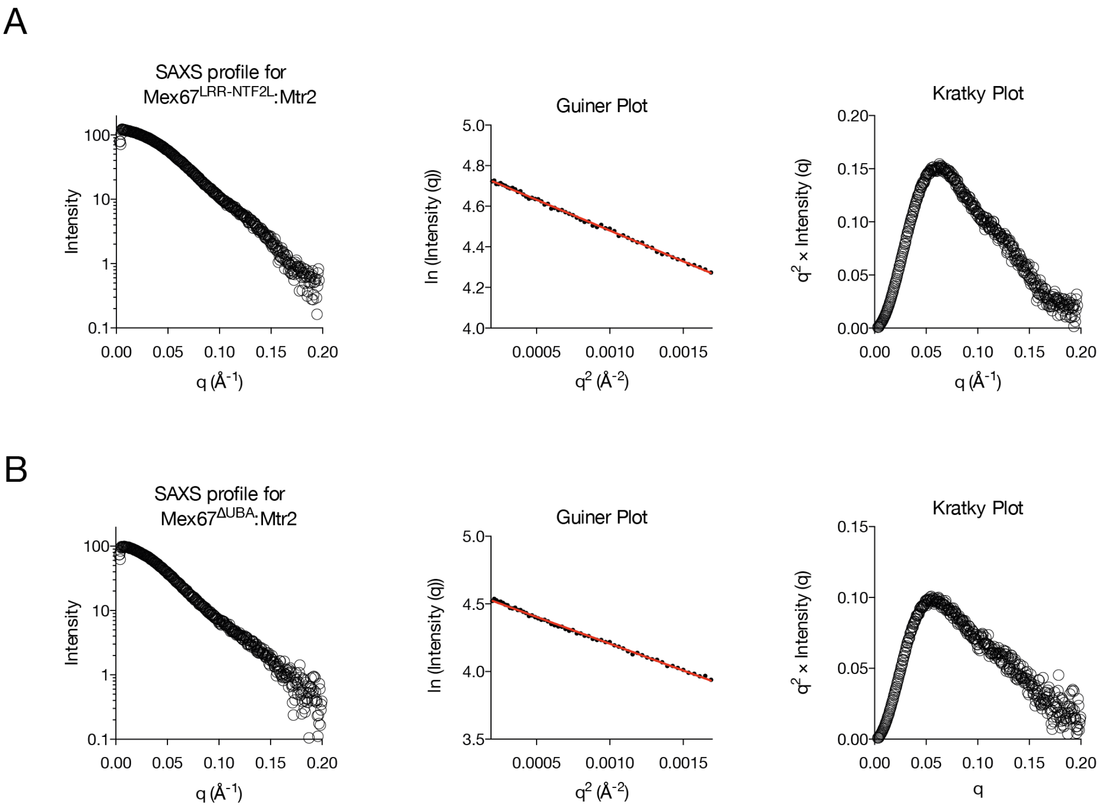

Supplementary Figure S1: The observed SAXS scattering, and the corresponding Guinier plot and Kratky plot for **(A)** Mex67$^{LRR-NTF2L}$:Mtr2 and **(B)** Mex67$^{\Delta UBA}$:Mtr2. In both cases, the Guinier plot (as generated by AUTORG using Primus) was linear, consistent with the samples used to obtain the profiles not bing affected by protein aggregation. Both Kratky plots display the characteristic bell-shaped curve suggesting that the protein is a folded molecule.

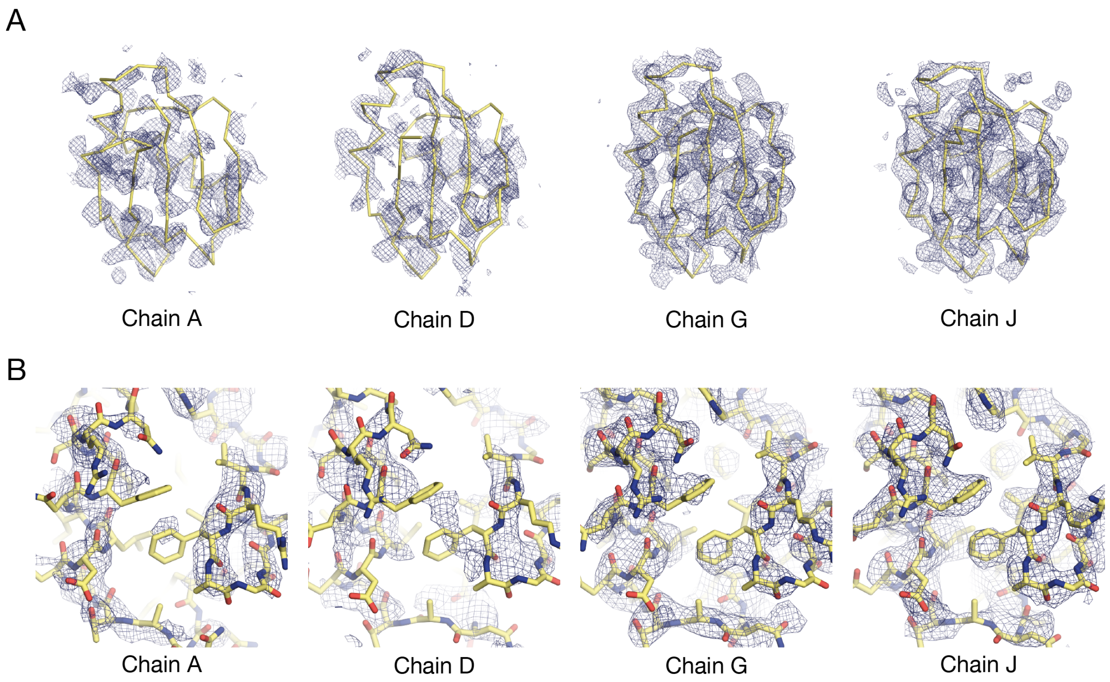

Supplementary Figure S2: (A) Simulated-annealed OMIT maps, contoured at 1 σ, calculated for the crystal structure of Mex67$^{\Delta UBA}$:Mtr2. The map generated around each RRM domain containing chain is shown. Continuous electron density was observed for chains G and J, but that for chains A and D was somewhat weaker. **(B)** Detailed view of the simulated-annealled OMIT map in the region between $\alpha 2$ and $\beta 4$ of the RRM domain.

A

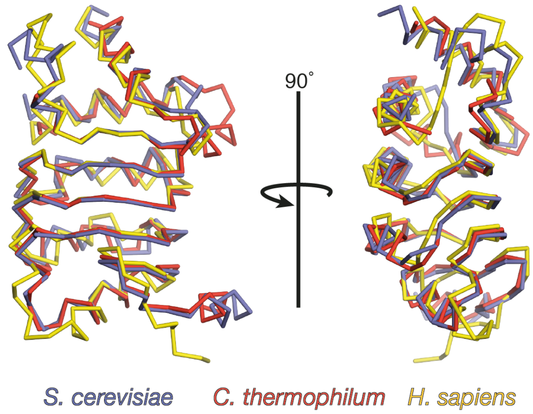

B

Structural alignment of the LRR domain

(Upper right: Cα-RMSD; lower left: Sequence Identity)

	*ct*Mex67-LRR	*sc*Mex67-LRR	*hs*NXF1-LRR
*ct*Mex67 LRR		0.85 Å (111)	1.5 Å (116)
*sc*Mex67 LRR	36 % (141)		1.8 Å (117)
*hs*NXF LRR	27 % (135)	29 % (145)	

C

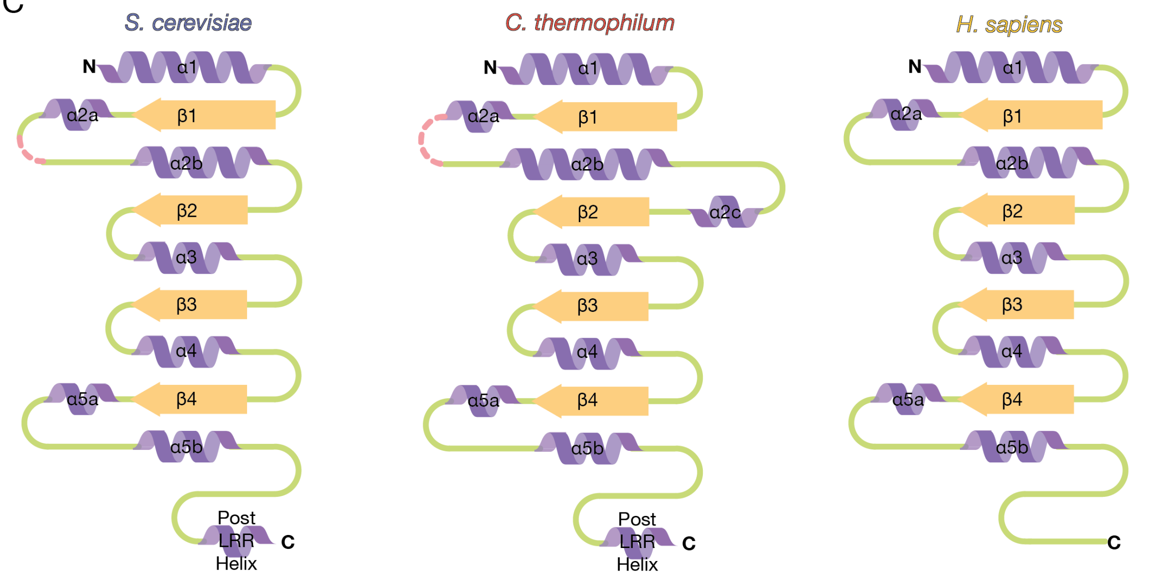

Supplementary Figure S3: (A) Structural alignment of the LRR domains from *S. cerevisiae* Mex67 (PDB ID: 4WWU, blue), *C. thermophilum* Mex67 (PDB ID: 4WP6, red), and *H. sapiens* NXF1 (PDB ID: 3RW6, yellow). **(B)** Table summarizing the structure based sequence identity and the Cα-RMSD between the three LRR domains shown in panel A. The numbers of residues used for the alignment are given in parentheses. The structure-based sequence alignments were calculated using the DaliLite server (1), whereas the Cα-RMSDs were calculated using the *super* command in PyMol using default settings. **(C)** Schematic illustration of the secondary structure elements present in the three LRR domains. Regions of disorder are depicted as broken red lines.

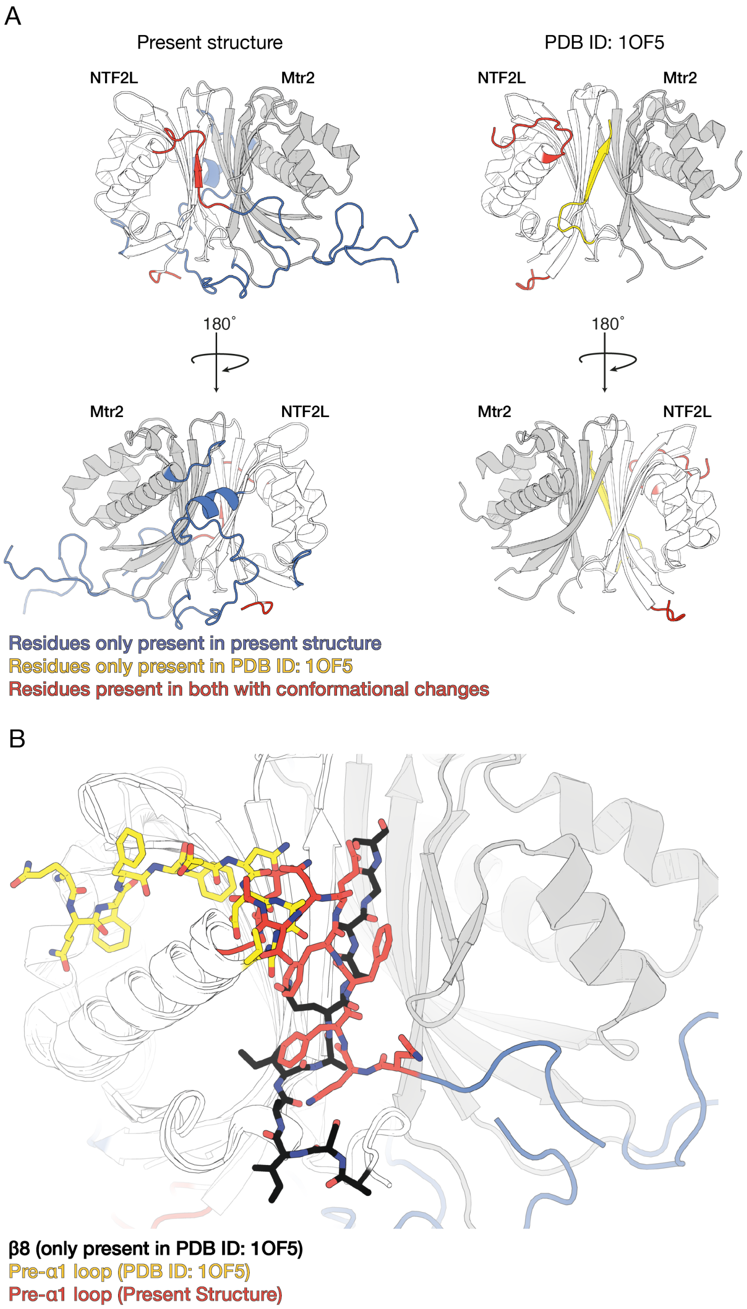

Supplementary Figure S4: (A) Overview of the similarities and differences observed between the NTF2L:Mtr2 region from Mex67:Mtr2 of the multi-domain structure (left) and the isolated domain (right, PDB ID: 1OF5). **(B)** Detailed view of the pre-α1 loop region obtained by aligning the two NTF2L:Mtr2 regions from panel A structurally. The β8 strand formed by non-native residues (black) observed in the isolated structure occupied the position observed for the pre-α1 loop (red). As a consequence of the interactions generated by the non-native β8 strand, the pre-α1 from the isolated domain structure is present in an alternative position (yellow).

A

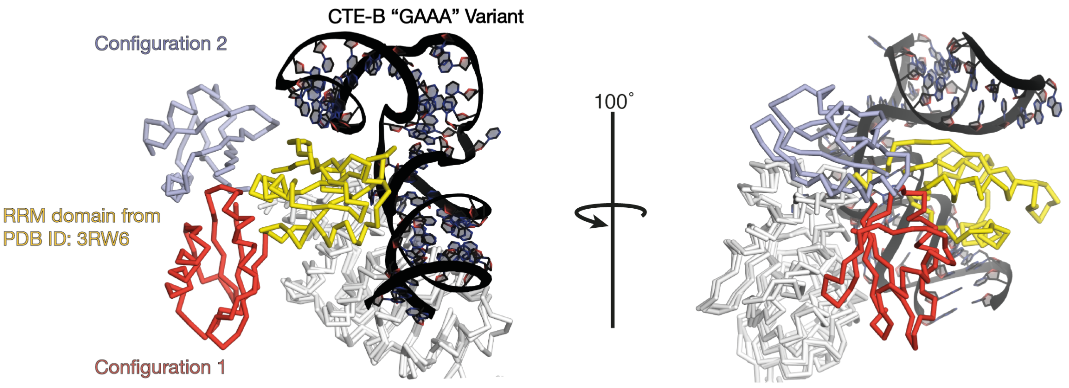

B

S. cerevisiae (sc) Mex67-RRM domain compared with *H. sapiens* (hs) NXF1-RRM domain (PDB ID: 3RW6)

```
                 :         :         :         :         |         :         :
scRRM   KISVRNWQNATMNDLINFISRNARVAVYDAHVEGPLVIGYVNSKAEAESLMKWNGVRFAGSNLKFELLDD
hsRRM   KITIPYGRKYDKAWLLSMIQSKCPFTPIEFHYENTRAQFFVEDASTASALKAVNYILDRERRISIIINSS
```

S. cerevisiae (sc) Mex67-LRR domain compared with *H. sapiens* (hs) NXF1-LRR domain (PDB ID: 3RW6)

```
                 :         :         :         :         |         :         :
scLRR   DTISFLRGVLLKRYDPQTKLLNLGALHSDPELIQKGVSTQSKMFPAMMKLASTEKSLIVESVNLADNQLK
hsLRR   EQVEQLKLIMSKRYDGSQQALDLKGLRSDPDLVQNID--RRSCMAATLRIIEEN---ELLSLNLSNNRLY

                 :         :         100       :         :         :         :
scLRR   DISAISTLAQTFPNLKNLCLANNQIFRFRSLEVWKNKFKDLRELLMTNNPITTDKLYRTEMLRLFPKLVV
hsLRR   RLDDMSSIVQKAPNLKILNLSGNELKSERELDKI-KGLK-LEELWLDGNSLCDTFTYISAIRERFPKLLR

                 |
scLRR   LDNVIVRDEQKLQTV
hsLRR   LDGHELPP---PIAF
```

Supplementary Figure S5: (A) Comparisons of the two configurations of the RRM domain from *sc*Mex67$^{\Delta UBA}$:Mtr2 (configurations 1 and 2, blue and red respectively) and the RRM domain from the structure of *hs*NXF1$^{RRM\text{-}LRR}$ in complex with CTE-B RNA (RRM domain in yellow, RNA in black). The position of the NXF1 RRM domain is quite different to that seen with Mex67 using the DaliLite server (1), but is probably determined by its binding to CTE-B RNA. **(B)** Structure based sequence alignment of the *S. cerevisiae* Mex67 and *H. sapiens* NXF1 RRM domains (top) and LRR domains (bottom). Residues implicated in binding CTE-B RNA (2) are highlighted.

SUPPLEMENTARY REFERENCES

1. Hasegawa, H. and Holm, L. (2009) Advances and pitfalls of protein structural alignment. *Curr. Opin. Struct. Biol.*, **19**, 341–8.

2. Teplova, M., Wohlbold, L., Khin, N.W., Izaurralde, E. and Patel, D.J. (2011) Structure-function studies of nucleocytoplasmic transport of retroviral genomic RNA by mRNA export factor TAP. *Nat. Struct. Mol. Biol.*, **18**, 990–8.
